# Role of Metabolic Genes in Blood Aluminum Concentrations of Jamaican Children with and without Autism Spectrum Disorder

**DOI:** 10.3390/ijerph13111095

**Published:** 2016-11-08

**Authors:** Mohammad H. Rahbar, Maureen Samms-Vaughan, Meagan R. Pitcher, Jan Bressler, Manouchehr Hessabi, Katherine A. Loveland, MacKinsey A. Christian, Megan L. Grove, Sydonnie Shakespeare-Pellington, Compton Beecher, Wayne McLaughlin, Eric Boerwinkle

**Affiliations:** 1 Division of Epidemiology, Human Genetics, and Environmental Sciences (EHGES), University of Texas School of Public Health at Houston, Houston, TX 77030, USA; Jan.Bressler@uth.tmc.edu (J.B.); Eric.Boerwinkle@uth.tmc.edu (E.B.); 2 Division of Clinical and Translational Sciences, Department of Internal Medicine, University of Texas McGovern Medical School at Houston, Houston, TX 77030, USA; 3 Center for Clinical and Translational Sciences (CCTS), University of Texas Health Science Center at Houston, Houston, TX 77030, USA; Meagan.R.Pitcher@uth.tmc.edu (M.R.P.); Manouchehr.Hessabi@uth.tmc.edu (M.H.); Mackinsey.A.Christian@uth.tmc.edu (M.A.C.); 4 Department of Child & Adolescent Health, The University of the West Indies (UWI), Mona Campus, Kingston 7, Jamaica; msammsvaughan@gmail.com (M.S.-V.); sydonniesp@gmail.com (S.S.-P.); 5 Human Genetics Center, University of Texas School of Public Health at Houston, Houston, TX 77030, USA; Megan.L.Grove@uth.tmc.edu; 6 Department of Psychiatry and Behavioral Sciences, University of Texas McGovern Medical School at Houston, Houston, TX 77054, USA; Katherine.A.Loveland@uth.tmc.edu; 7 Department of Basic Medical Sciences, The University of the West Indies, Mona Campus, Kingston 7, Jamaica; compton.beecher@uwimona.edu.jm (C.B.); wayne.mclaughlin@uwimona.edu.jm (W.M.); 8 Caribbean Genetics (CARIGEN), The University of the West Indies, Mona Campus, Kingston 7, Jamaica

**Keywords:** aluminum, Autism Spectrum Disorder (ASD), glutathione S-transferase (GST) genes, detoxification, interactions

## Abstract

Aluminum is a neurotoxic metal with known health effects in animals and humans. Glutathione-S-transferase (GST) genes and enzymes play a major role in detoxification of several heavy metals. Besides a direct relationship with oxidative stress; aluminum decreases GST enzyme activities. Using data from 116 Jamaican children; age 2–8 years; with Autism Spectrum Disorder (ASD) and 116 sex- and age-matched typically developing (TD) children; we investigated the association of polymorphisms in three GST genes (*GSTP1*; *GSTM1*; and *GSTT1*) with mean blood aluminum concentrations in children with and without ASD. Using log-transformed blood aluminum concentration as the dependent variable in a linear regression model; we assessed the additive and interactive effects of ASD status and polymorphisms in the three aforementioned GST genes in relation to blood aluminum concentrations. Although none of the additive effects were statistically significant (all *p* > 0.16); we observed a marginally significant interaction between *GSTP1* Ile105Val (rs1695) and ASD status (*p* = 0.07); even after controlling for parental education level and consumption of avocado; root vegetables; and tuna (canned fish). Our findings indicate a significantly lower (*p* < 0.03) adjusted geometric mean blood aluminum concentration for TD children who had the Val/Val genotype (14.57 µg/L); compared with those with Ile/Ile or Ile/Val genotypes who had an adjusted geometric mean of 23.75 µg/L. However; this difference was not statistically significant among the ASD cases (*p* = 0.76). Our findings indicate that ASD status may be a potential effect modifier when assessing the association between *GSTP1* rs1695 and blood aluminum concentrations among Jamaican children. These findings require replication in other populations.

## 1. Introduction

Aluminum (Al) is found naturally in silicates, cryolite, and bauxite rocks and is the third most abundant element in the earth’s crust [1,2,3]. Aluminum has no known biological role [4], but has an important biogeochemical cycle [1] and at high concentrations can have widespread environmental effects [1,5] and cause toxicity in a variety of living organisms, including microbes [4,6], plants [7], fish [5], and mammals [8]. High levels of aluminum in acidic soil and water can limit growth of plants and aquatic organisms [9]. At the same time, accumulation of aluminum in plants and freshwater invertebrates is a potential route for aluminum exposure in mammals [5] as well as for accumulation with repeated exposure [10]. Routes of human exposure to aluminum include food [3] or water through the digestive tract, skin, and occupational inhalation [2,3,11]. Some studies suggest that aluminum exposure in humans is associated with utilization of cookware and food packaging materials made from aluminum [12,13]. In addition, aluminum is found in some consumer products such as antacids (aluminum hydroxide), astringents, food additives (aluminum oxides), antiperspirants, fuel additives, explosives, propellants, and cosmetics [3,14]. Aluminum is excreted from the human body through the feces and urine [2,14].

As a neurotoxic agent and destabilizer of cell membranes, aluminum is associated with several neurodegenerative diseases [15,16,17]. Moreover, recent findings have implicated astrocytes, which are responsible for the physical blood-brain barrier [18] and regulate glutamate and gamma aminobutyric acid (GABA) neurotransmission [19] as the principal target of aluminum toxicity [18,20]. Other studies speculated that aluminum diminishes the ability of astrocytes to protect neurons from glutamate toxicity [18]. On the other hand, GABA level, glutamate/GABA and glutamine/glutamate ratios are reported to be significantly lower in children with Autism Spectrum Disorder (ASD) compared to typically developing (TD) controls [21,22]. Another study reported associations between reduced GABA level, neuroinflammation, and glutamate excitotoxicity [23]. A study that involved 20 children with ASD (aged 3–15 years) and 20 age- and sex-matched TD controls from Saudi Arabia reported a significant association between ASD status and levels of nine biomarkers including levels of glutathione (GSH), glutamate excitotoxicity, and impaired detoxification [24].

Due to its high reactivity, Al^3+^ is able to interfere with enzymatic activities in key metabolic pathways. For example, two of the enzymes that constitute the Krebs cycle are activated by aluminum [15]. The toxic effects of aluminum may also include interference with secondary messenger systems [25]; competition with Mg^2+^ for phosphate sites in critical biological enzymes, such as ATPase [8,26]; and enhancement of iron-induced reactive oxygen species production and oxidative stress [27]. Additionally, a well-known cause of aluminum toxicity in living organisms is by induction of oxidative stress [8,25,28,29].

Besides a direct relationship with oxidative stress accumulation, aluminum inhibits biological oxidative stress management systems by interfering with the glutathione S-transferase (GST) detoxification system [17,30]. GSTs are multi-gene isoenzymes that are encoded by three separate families of genes, including cytosolic, microsomal, and mitochondrial transferases [31,32,33,34,35], which are involved in the cellular detoxification of both xenobiotic and endobiotic compounds [31,32,36]. The human GST gene superfamily comprises eight classes: alpha, kappa, mu, omega, pi, theta, sigma, and zeta [34]. Glutathione S-transferase pi (*GSTP1*), glutathione S-transferase mu (*GSTM1*), and glutathione S-transferase theta (*GSTT1*) play important roles in detoxification of xenobiotics [37] and polymorphisms in these genes may affect biologic responses to heavy metals. For example, people with a double deletion of *GSTM1* and *GSTT1* have higher mercury concentrations in hair than other individuals, suggesting that the epistatic effect of this double deletion is a risk factor for increased susceptibility to mercury toxicity [38,39].

GST genes encode enzymes that catalyze the conjugation of the reduced form of GSH to xenobiotics, such as heavy metals, thereby reducing the toxic effects and promoting excretion of the conjugated form of the xenobiotic [34,35]. In three studies, rodents treated with aluminum have reduced amounts of GSH [17,30,40]. The aluminum-associated GST/GSH effects observed in animal models have been reproduced in human studies. For example, a study of industrial workers noted that people with the highest levels of aluminum in urine also have low GST enzymatic activity in erythrocytes [41].

Recent findings from our Jamaican study of children with ASD and TD children revealed that the relationship between *GSTP1* and blood arsenic concentrations varied by ASD status (TD or ASD) in an interactive model [42] In addition, we identified a significant gene-gene interaction between *GSTP1* and *GSTT1* in relation to ASD, where there was a significantly higher odds of ASD for children who were heterozygous for the *GSTP1* Ile105Val polymorphism and had the *GSTT1* null genotype (Matched Odds Ratio (MOR) = 2.97, 95% CI (1.09, 8.01), *p* = 0.03) [43]. Furthermore, we found a significant gene-environment interaction between *GSTP1* and blood manganese concentrations (BMC) indicating that among children who had the Ile/Ile genotype for *GSTP1*, those with BMC ≥ 12 µg/L had about four times higher odds of ASD than those with BMC < 12 µg/L (*p* = 0.03) [44].

Although a number of studies have examined the developmental effects of toxicity of aluminum in rats and mice [2], limited data are available on the toxicity of aluminum in children [2]. Higher levels of aluminum were observed in the hair [45,46,47] and urine [48] of children with ASD, compared to children without ASD. One study from Egypt additionally found that the level of aluminum in children’s hair was positively correlated with the use of aluminum pans in the home [45]. However, some studies found no association between aluminum level in hair [49,50] and aluminum level in blood and urine of children with ASD [51]. Jamaica’s soil has higher levels of aluminum than most countries around the world, with the highest aluminum concentrations near the main bauxite-mining belt and in small areas on the east and the west coasts [52]. The main objective of this study was to investigate the role of polymorphisms in three GST genes (*GSTP1*, *GSTM1*, and *GSTT1*) in relation to blood aluminum concentrations using data from a sex- and age-matched case-control study of ASD and TD Jamaican children, ages 2–8. Furthermore, we investigated whether ASD status is an effect modifier when exploring the association between GST gene polymorphisms and blood aluminum concentrations.

## 2. Materials and Methods

### 2.1. General Description

The Jamaican Autism Study is an age- and sex-matched case-control study of Jamaican children between 2 and 8 years of age that were enrolled during December 2009–May 2012. The overall goal of the Jamaican Autism Study is to investigate if environmental exposures to aluminum, arsenic, mercury, lead, cadmium, and manganese play a role in ASD, and to assess the role of polymorphisms in three GST genes (*GSTT1*, *GSTM1*, and *GSTP1*) and their interactions with these heavy metals in relation to ASD status. Detailed information regarding the recruitment and assessment of ASD cases and TD controls was reported earlier [53,54]. In brief, children included in the University of the West Indies (UWI) Jamaica Autism Database, who were previously identified as being at risk for an ASD based on Diagnostic Statistical Manual of Mental Disorders (DSM-IV-TR) criteria [55] and the Childhood Autism Rating Scale (CARS) [56], were invited to participate for reassessment of their ASD status for this study. We administered the Autism Diagnostic Observation Schedule (ADOS) [57] and the Autism Diagnostic Interview-Revised (ADI-R) [58] to confirm the diagnosis of ASD. For ascertainment of ASD, we used standard algorithms developed for scoring ADOS and ADI-R and established cut-off points. Each ASD case was confirmed based on both ADI-R and all three domains in ADOS. For each confirmed ASD case, an age- and sex-matched TD control was identified from schools and well-child clinics. The criteria for matching required that the age of the control child be within six months of the matched ASD case. For ascertainment of TD, we administered the Lifetime Form of the Social Communication Questionnaire (SCQ) [59] to the parents/guardians of potential TD control children to rule out symptoms of ASD. We set the criteria for including children in the TD control group as having a SCQ score of 0–6. This cut-off point of 6 is one standard deviation above the mean SCQ score of TD school children [60]. A SCQ score more than six indicated a possible developmental delay and the child was referred for further evaluation.

We also administered a pre-tested questionnaire to the parents/guardians of both ASD cases and TD controls to collect information about their demographic and socioeconomic status (SES), such as ownership of a car by the family, parental education levels, and potential exposure to heavy metals through drinking water sources and food, especially the types and frequency of fruits, vegetables, and seafood consumed by the children on a weekly basis. The types of seafood, fruits, and vegetables were classified into different categories, based on their characteristics and species. For example, the types of fruits and vegetables were classified into the following categories: (1) two classes of root vegetables ((yam, sweet potato, or dasheen) and (carrot, or pumpkin)); (2) three classes of leafy vegetables ((lettuce), (callaloo, broccoli, or pak choi), and (cabbage)); (3) legumes (string beans); and (4) three different fruits (tomatoes, ackee, or avocado). For analysis, we categorized the frequency of food consumption into two levels (consumed and never consumed). Details regarding the categories of fruits and vegetables were reported earlier [53].

About 5 mL of venous whole blood was drawn from each child. Samples for trace metals analysis (2 mL) were stored at −20 °C while the remaining samples for genetic analysis (3 mL) were stored at −80 °C at UWI in Kingston, Jamaica. In addition, 2 mL of saliva was also collected from each child. Whole blood samples for trace metal analysis were shipped to the Michigan Department of Human Health Services (MDHHS), and the whole blood and saliva samples for genetic analysis were shipped to the Human Genetics Center at the University of Texas Health Science Center at Houston School of Public Health. All parents/guardians provided written informed consent. The study was conducted in accordance with the Declaration of Helsinki, and the protocol was approved by the Institutional Review Boards (IRBs) of the University of Texas Health Science Center at Houston (UTHealth) and UWI, Mona Campus, in Kingston, Jamaica (Project identification code: HSC-SPH-09-0059). The data presented herein represent analysis of 116 1:1 matched case-control pairs for whom we had complete data.

### 2.2. Assessment of Aluminum Exposures

Aluminum exposure can be assessed using various biological specimens including blood, hair, and urine depending on duration of exposure [2]. Greater than 95% of aluminum is excreted from the human body through the kidneys. Steady state blood aluminum concentrations in serum and in whole blood are similar [3]. Occupational aluminum exposure could result in a greater increase in urinary than in plasma aluminum concentrations [3]. Depending on the type and route of exposure, clearance of aluminum from the body is estimated to vary from hours to years [3]. Although blood is considered a more reliable method for measuring recent aluminum exposures, blood aluminum concentrations may not accurately reflect the total body burden of aluminum and most literature recommends assessing aluminum in urine [2,3]. In fact brain, lung, and bone measurements may reveal much higher levels of aluminum than are found in the blood [61]. On the other hand, in extreme exposures, aluminum in hair is not associated with concentrations of aluminum in serum or bone [62,63] and is unrelated to the dietary intake [64].

Although sources of exposure to aluminum include drinking water and food [3], the extent of aluminum absorption depends on a number of factors, including pH (for aluminum speciation and solubility), bioavailability, and diet [65]. Foods that are naturally high in aluminum include potatoes, spinach, and tea. Processed dairy products, flour, and infant formula may be high in aluminum if they contain aluminum-based food additives [65]. Most studies do not consider the speciation of aluminum, as the assessment of exposure from both drinking water and food is not well characterized [65]. However, it is important to note that aluminum is not bioaccumulated to a significant extent in aquatic organisms [2]. In addition, currently there is a lack of data to support a correlation between aluminum levels measured in different biological samples [66].

Since the Jamaican community has continuous exposure to aluminum through various sources including soil, water, and the food chain, possibly through fruits and vegetables, we considered aluminum in whole blood a reliable biomarker for aluminum exposure. In this study, blood aluminum concentrations were assessed by the Trace Metals Lab at MDHHS, which is recognized by the Centers for Disease Control and Prevention (CDC). The Trace Metals Lab is certified for testing toxic metals in blood and urine by the College of American Pathology (CAP). MDHHS followed a fully validated protocol for analyzing aluminum in blood samples with a detection limit of 5.0 µg/L. All samples were diluted and analyzed on a PerkinElmer Nexion 300D inductively-coupled plasma mass spectrometer (PerkinElmer, Waltham, MA, USA). Six out of 232 children in this study had blood aluminum concentrations below the limit of detection (LoD). In data analyses, these study participants were assigned a blood aluminum concentration of 2.5 µg/L (midpoint between 0.0 and 5.0 µg/L).

### 2.3. Genetic Analysis

All procedures used for genetic analysis were carried out as previously described [42]. In brief, plasma, buffy coat, and red blood cells were isolated from whole blood samples collected for each participant at UWI, and stored at −80 °C for future use. Saliva was collected with either the Oragene Discover DNA Collection Kits for Research (DNA Genotek Inc., Kanata, ON, Canada) or the Oragene Discover for Assisted Collection (DNA Genotek). Frozen blood specimens or saliva samples at ambient temperature were shipped from UWI to the UTHealth School of Public Health Human Genetics Center. Genomic DNA was isolated from buffy coat using the Gentra PUREGENE Blood Kit (Qiagen, N.V., Venlo, The Netherlands), or if a buffy coat was not available, from saliva with the Gentra PUREGENE DNA Purification Kit (Qiagen protocol 400244 Rev A., Kanata, ON, Canada) [67]. *GSTT1* and *GSTM1* genotypes were measured using a multiplex polymerase chain reaction (PCR) [68]. The *GSTP1* Ile105Val polymorphism (rs1695) was genotyped using the TaqMan Drug Metabolism SNP Genotyping Assay C_3217198_20 using the thermal cycling parameters recommended by the manufacturer (Thermo Fisher Scientific, Waltham, MA, USA). Allele detection was performed using the ABI Prism 7700 Sequence Detection System (Thermo Fisher Scientific).

### 2.4. Statistical Analysis

Descriptive analyses were conducted to compare demographic and SES characteristics of ASD cases and TD controls. We also assessed mean blood aluminum concentrations for children with and without ASD. Since the distribution of blood aluminum concentrations was skewed, the data were transformed using the natural logarithm (ln) to produce an approximately normal distribution. The means of the log transformed blood aluminum concentrations were transformed back to their original scale (i.e., µg/L) by applying the natural exponential function, herein called geometric means.

The PCR assay does not distinguish between a normal homozygote (I/I) and a heterozygote (I/D) for the *GSTM1* and *GSTT1* genes, so we used a recessive model with the genotype represented as a binary variable: I* and DD. Three genotypes, Ile/Ile, Ile/Val and Val/Val, were available for the *GSTP1* gene. We analyzed the *GSTP1* genotypes using different genetic models, including the recessive (Ile/* vs. Val/Val) and full models (Ile/Ile, Ile/Val and Val/Val). For the *GSTP1* genotypes, we tested accordance with Hardy–Weinberg equilibrium expectations using the chi-square test in the TD control group.

In this study, we used General Linear Models (GLMs) with the log-transformed blood aluminum concentrations as the dependent variable to investigate the role of genetic variants of three GST genes in blood aluminum concentrations, while controlling for potential confounding variables. In order to minimize any potential effects of multicollinearity due to a high correlation between maternal and paternal education levels, we created a binary variable indicating whether both parents had education up to high school or at least one of the parents obtained education beyond high school. In all GLMs, we also controlled for the clustering effect of matching by including an appropriate number of dummy variables that represented the matched pairs (e.g., 105 dummy variables for 106 matched pairs used for genetic analysis and 115 dummy variables for 116 matched pairs used for analyses that did not utilize genetic data). In multivariable GLMs, we assessed interactions between each of the three GST genes and ASD status while controlling for potential confounding variables that included parental education levels, and consumption of root vegetables (yam, sweet potato, or dasheen), avocado, and tuna (canned fish). Because we found a significant interaction between ASD status and *GSTP1* genotypes in relation to blood aluminum concentrations, we used the CONTRAST statement in PROC GLM in SAS [69] to test whether there was a significant difference in geometric mean blood aluminum concentrations between individuals with the three *GSTP1* genotypes, separately for ASD cases and TD controls. Similarly, we tested whether there was a significant difference in geometric mean blood aluminum concentrations between ASD cases and TD controls after stratifying by *GSTP1* genotype. We calculated unadjusted and adjusted geometric mean blood aluminum concentrations for both groups of children (ASD and TD) with different *GSTP1* genotypes. All analyses were performed using the SAS software [70].

## 3. Results

The mean age of children with ASD was 67.5 months and the mean age for TD children was 68.4 months. As expected, about 85.3% of the ASD cases were male, and consequently, the same percentage of TD controls were male. Nearly all of the ASD cases (93.1%) and TD controls (99.1%) were Afro-Caribbean. Similarly, 96.6% of mothers and 95.7% of fathers were Afro-Caribbean. Our data indicated a frequency of 25.5% and 20.8% for the *GSTM1* and *GSTT1* null genotype, respectively, for TD children. There was no significant deviation from Hardy–Weinberg equilibrium for the *GSTP1* genotype in the TD controls (*p* = 0.70). In addition, there were no significant differences between ASD cases and TD controls with respect to the genotype frequencies of *GSTM1*, *GSTP1*, and *GSTT1* (all *p* > 0.16). The arithmetic mean blood aluminum concentration for children with ASD was 30.9 µg/L, the mean blood aluminum concentration for TD children was 36.9 µg/L. Demographic and other characteristics of children and their parents by ASD case status are presented in Table 1.

A comparison of dietary consumption between ASD cases and TD controls revealed that a significantly lower proportion of ASD cases reported eating avocado (Matched Odds Ratio (MOR) = 0.18, 95% CI (0.09, 0.35), *p* < 0.01) and root vegetables (yam, sweet potato, or dasheen) (MOR = 0.48, 95% CI (0.25, 0.93), *p* = 0.03). In addition, a significantly lower proportion of ASD cases reported consumption of other fruits and vegetables. Comparisons of other variables related to dietary consumption between children with and without ASD are displayed in Table 2.

We compared geometric mean blood aluminum concentrations between children who had different levels and types of exposures. In the univariable analysis, there was no significant association between blood aluminum concentrations and *GSTM1* and *GSTT1* (*GSTM1* (*p* = 0.31), *GSTT1* (*p* = 0.98), or *GSTP1* polymorphisms (all pairwise comparisons *p* > 0.10)). Additionally, there were no significant associations between blood aluminum concentrations and the consumption of seafood or fruits and vegetables. There was also no significant association between blood aluminum concentrations and ASD status (geometric mean blood aluminum concentrations for ASD group = 21.49 µg/L vs. 20.95 µg/L for the TD control group, *p* = 0.78). Associations of other exposure variables with blood aluminum concentrations are reported in Table 3.

**Figure 1:**
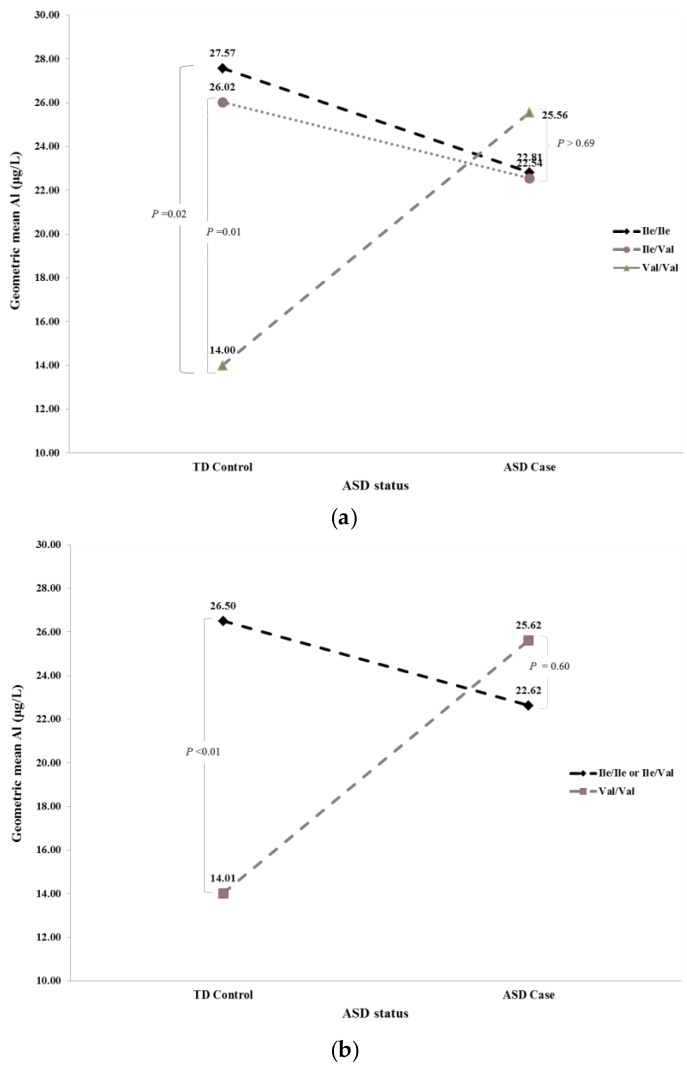
(**a**) Unadjusted geometric blood aluminum concentrations based on result from Full Model in Table 4; and (**b**) unadjusted geometric blood aluminum concentrations based on result from Recessive Model in Table 4.

In an unadjusted recessive multivariable model where we assessed the relationship between *GSTP1* genotype and blood aluminum concentrations within the ASD case and TD control groups, we identified a significant interaction between ASD status and *GSTP1* genotype (*p* < 0.02) in relation to blood aluminum concentrations. Further analysis of this interaction revealed that while there was no significant association between *GSTP1* rs1695 and blood aluminum concentrations within the ASD case group, there was a significant association between *GSTP1* rs1695 and blood aluminum concentrations within the TD control group. For example, in the recessive model, ASD cases with either an Ile/Ile or Ile/Val genotype had a geometric mean blood aluminum concentration of 22.62 µg/L, not significantly different (*p* = 0.60) from 25.62 µg/L for ASD cases with the Val/Val genotype. However, in the TD control group, we observed a geometric mean blood aluminum concentration of 14.01 µg/L for children with the Val/Val genotype significantly lower (*p* < 0.01) than the geometric mean of 26.50 µg/L among those with Ile/Ile or Ile/Val genotypes. Similar findings were observed for the TD control group in the adjusted recessive model. Specifically, we observed a significantly lower (*p* < 0.03) geometric mean blood aluminum concentration of 14.57 µg/L for children who had the Val/Val genotype, compared with a geometric mean of 23.75 µg/L among those with either Ile/Ile or Ile/Val genotypes. Details regarding the comparison of the unadjusted and adjusted geometric blood aluminum concentrations between individuals with different *GSTP1* genotypes by ASD status are shown in Table 4. Graphical illustrations of unadjusted geometric blood aluminum concentrations between those with different *GSTP1* genotypes by ASD status based on the full and recessive models are provided in Figure 1a,b.

**Figure 2:**
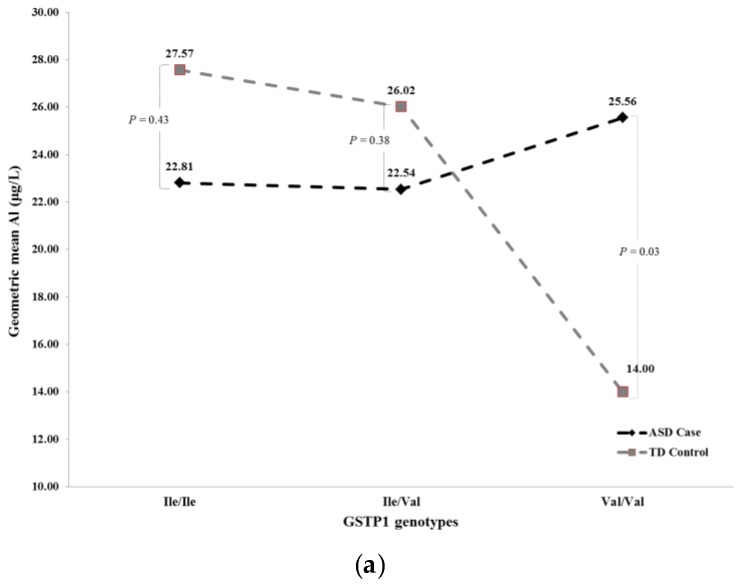

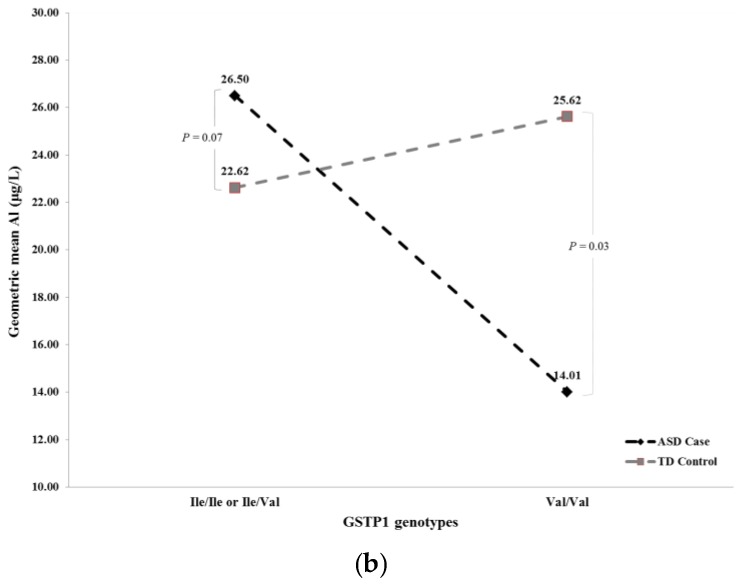
(**a**) Unadjusted geometric blood aluminum concentrations based on result from Full Model in Table 5; and (**b**) unadjusted geometric blood aluminum concentrations based on result from Recessive Model in Table 5.

Further analysis of the significant interaction between ASD status and *GSTP1* genotype in relation to blood aluminum concentrations revealed that while there was no significant association between ASD status and blood aluminum concentrations in children with the Ile/Ile and Ile/Val genotype (*p* = 0.43 and *p* = 0.38, respectively), there was a significant (*p* = 0.03) association between ASD status and blood aluminum concentrations in children with the Val/Val genotype. Similarly, in the recessive model, ASD children with the Val/Val genotype had a geometric mean blood aluminum concentrations of 25.62 µg/L, significantly higher (*p* = 0.03) than that observed for TD control children with the same Val/Val genotype (14.01 µg/L). However, when we adjusted for potential confounders, including parental education levels and consumption of root vegetables (yam, sweet potato, or dasheen), avocado, and tuna (canned fish), these associations became marginally significant (*p* = 0.07) in the analysis using both the full and recessive models. Details regarding the comparison of the unadjusted and adjusted geometric blood aluminum concentrations between ASD cases and TD controls by *GSTP1* genotype are shown in Table 5. Graphical illustrations of unadjusted geometric blood aluminum concentrations between ASD cases and TD control groups by *GSTP1* genotype based on the full and recessive models are provided in Figure 2a,b.

## 4. Discussion

### 4.1. Blood Aluminum Concentrations in Jamaican Children

In this research, to our knowledge, we are the first to assess and report distributions of blood aluminum concentrations for Jamaican children with and without ASD (2–8 years). The arithmetic mean blood aluminum concentration for children with ASD was 30.9 µg/L and the mean blood aluminum concentration for TD children was 36.9 µg/L. Data regarding blood aluminum concentrations in children are limited [2], and, to our knowledge, published reference ranges are not established for children. However, for healthy individuals the Agency for Toxic Substances and Disease Registry (ATSDR) considers a serum aluminum concentration of 1.00–3.00 µg/L as “normal” [2,71,72]. Therefore, it will not be possible to reliably estimate the proportion of Jamaican children with abnormal blood aluminum concentrations. On the other hand, aluminum toxicity is defined as a plasma aluminum concentration of >500 μg/L [2,71,72]. In our sample, the highest blood aluminum concentration was 221 μg/L. Therefore, none of the children in our sample had blood aluminum concentrations at the defined toxicity level.

Other studies have reported blood aluminum concentrations of children living in other countries. For example, a study from Riyadh, Saudi Arabia that involved 533 girls (6–8 years) reported a mean serum aluminum concentration of 23.21 μg/L (range 5.98–206.93 μg/L) [73]. Another study conducted in Romania during 2006–2007 involved two samples of children (8–12 years), one sample from Bucharest (*N* = 37) and the other sample from Pantelimon (*N* = 46), a city near a metal-processing plant. The Romania study reported geometric mean blood aluminum concentrations of 36 μg/L and 49 μg/L for Bucharest and Pantelimon, respectively [74]. While the mean blood aluminum concentration of Jamaican children is similar to that of children from Bucharest, it is higher than the mean serum aluminum concentration reported for children from Riyadh, Saudi Arabia. We acknowledge that the aluminum concentrations reported by the study from Saudi Arabia were measured in serum, which are shown to be similar to that of whole blood in the steady state [3].

### 4.2. Blood Aluminum Concentrations and ASD

Although we did not find a significant difference between the geometric mean blood aluminum concentrations of the ASD and TD control groups, in a recessive and in a full model that included *GSTP1*, ASD status, and their interaction term (i.e., *GSTP1* × ASD) status we found significant interactions between ASD status and the *GSTP1* Ile105Val polymorphism in relation to blood aluminum concentrations in both recessive and full models. Further analysis of the significant interaction between ASD status and the *GSTP1* variant in relation to blood aluminum concentrations revealed that while there was no significant difference between ASD and TD children with respect to blood aluminum concentrations in children with the Ile/Ile and Ile/Val genotypes, there was a significant association between ASD status and blood aluminum concentrations in children with the Val/Val genotype. Similarly, in the recessive model, ASD children with the Val/Val genotype had a geometric mean blood aluminum concentration of 25.62 µg/L, significantly higher than 14.01 µg/L for TD control children with the same Val/Val genotype. However, in multivariable analysis when we also added potential confounders, including parental education levels and consumption of root vegetables (yam, sweet potato, or dasheen), avocado, and tuna (canned fish), these associations became marginally significant in both full and recessive models. To our knowledge, we are the first to report an interactive association of ASD status and *GSTP1* rs1695 in relation to blood aluminum concentrations of Jamaican children.

Other studies have reported higher levels of aluminum in the hair [45,46,47] and urine [48] of children with ASD compared to children without ASD. Although it appears that our results contrast with results from other studies, the difference could be due to the differences in methods of assessment of aluminum concentrations; we determined the concentration of aluminum in blood while these other studies assessed aluminum concentrations in hair and urine. Some studies investigated whether there is a correlation between hair and serum aluminum concentrations, but none found any significant associations [62,63,75]. Another major difference between our study and these previous studies is that when we compared blood aluminum concentrations of children with and without ASD, we accounted for a significant interaction between the *GSTP1* variant and ASD status and also controlled for potential confounders. Specifically, in the multivariable GLM, we controlled for potential confounding by adjusting for parental education levels and the consumption of root vegetables (yam, sweet potato, or dasheen), avocado, and tuna (canned fish). However, none of these other studies adjusted their results by such factors.

### 4.3. Role of GST Genes in Blood Aluminum Concentrations of Jamaican Children with and without ASD

Another unique aspect of our study is the availability of genetic data related to *GSTT1*, *GSTM1*, and *GSTP1* genotypes. These data allowed assessment of associations between genetic variation in *GSTT1*, *GSTM1*, and *GSTP1* and aluminum concentrations in blood. In an additive model, we did not find a significant association between the three GST polymorphisms and aluminum concentrations in blood. However, as mentioned earlier, we found a significant interaction between *GSTP1* genotype and ASD status (*p* < 0.02) in relation to aluminum concentration in blood using an unadjusted recessive multivariable model. This finding indicates that the association between *GSTP1* rs1695 and blood aluminum concentrations varies by ASD status. Specifically, we did not find a significant difference between geometric mean blood aluminum concentrations when comparing children with the Val/Val genotype and the Ile/Ile or Ile/Val genotypes for ASD cases. However, in the TD control group we observed a significantly lower (*p* < 0.01) geometric mean blood aluminum concentration for children who had the Val/Val genotype (14.01 µg/L), compared with the geometric mean blood aluminum concentration among children with Ile/Ile or Ile/Val genotypes (26.50 µg/L). Similar findings were observed for the TD control group in the adjusted recessive models that controlled for covariates. Specifically, after controlling for parental education levels and the consumption of root vegetables (yam, sweet potato, or dasheen), avocado, and tuna (canned fish), we observed a significantly lower (*p* < 0.03) geometric mean blood aluminum concentration for children who had the Val/Val genotype (14.57 µg/L), compared with the geometric mean blood aluminum concentration among children with the Ile/Ile or Ile/Val genotypes (23.75 µg/L). A possible biological explanation for our finding that geometric mean blood aluminum concentrations in TD control children with the Val/Val genotype is lower than that of TD children with Val/Ile or Ile/Ile genotypes is that Val/Val homozygotes may detoxify aluminum more efficiently than those with other genotypes. This mechanism for detoxifying aluminum may be less efficient in ASD children with the same Val/Val genotype, leading to higher geometric blood aluminum concentrations compared to TD children. However, we did not find any previous literature supporting these hypotheses. To our knowledge, we are the first to report ASD status as an effect modifier when assessing the association between *GSTP1* rs1695 and blood aluminum concentrations. Confirmation of this finding in other populations is warranted.

## 5. Limitations

We acknowledge several limitations in this study. First, since the control children for this study were selected to match the ASD cases by sex and age from the Kingston area, they may not represent a random sample from the population of all children in Jamaica. Additionally, our controls belonged to a lower SES group than our ASD cases. Therefore, the findings reported in this study may not be generalizable to populations other than that in which the samples were selected. Although our analyses for food consumption (fruits, vegetables, and seafood) were conducted under the assumption that most products were grown and caught locally, we acknowledge that some participants may have consumed foods imported from other locations; however, we did not assess this possibility in the food frequency questionnaire. In addition, it is possible that our analysis did not account for unmeasured confounding variables that may have a strong correlation with blood aluminum concentrations and *GSTP1* rs1695 in the ASD group. We also acknowledge that it is possible that the observed association between *GSTP1* rs1695 and blood aluminum concentrations may not necessarily represent an effect of the sequence variant if it is in linkage disequilibrium with a true causal polymorphism that was not measured in this study. Finally, we acknowledge that the *p*-values reported in this study were not adjusted to account for multiple comparisons.

## 6. Conclusions

In this study, we reported mean blood aluminum concentrations for Jamaican children that could serve as baseline data for exposure to aluminum in Jamaican children, age 2–8 years. Specifically, the mean blood aluminum concentration was 36.87 µg/L for the TD Jamaican children. In addition, based on multivariable analysis that controlled for potential confounding by adjusting for parental education levels and consumption of root vegetables (yam, sweet potato, or dasheen), avocado, and tuna (canned fish), we reported that ASD status may serve as an effect modifier of the association between *GSTP1* rs1695 and blood aluminum concentrations of Jamaican children. However, it is difficult to provide a clear biological explanation for this finding. Future research should focus on better understanding this finding from our study.

## Figures and Tables

**Table 1:** Characteristics of children and their parents by ASD case status (116 matched pairs).

Variables	Categories	ASD Case (*n* = 116) *N* (%)	TD Control (*n* = 116) *N* (%)	*p*-Value *
Child’s sex	Male	99 (85.3)	99 (85.3)	1.00
Child’s age (months)	Age < 48	22 (19.0)	19 (16.4)	0.29
	48 ≤ age < 72	51 (44.0)	52 (44.8)	
	Age ≥ 72	43 (37.0)	45 (38.8)	
Child’s race	Afro-Caribbean	108 (93.1)	115 (99.1)	0.19
Maternal age ^a^ (at child’s birth)	<35 years	87 (75.0)	100 (99.1)	<0.01
	≥35 years	29 (25.0)	11 (9.0)	
Paternal age ^b^ (at child’s birth)	<35 years	57 (50.9)	78 (72.9)	<0.01
	≥35 years	55 (49.1)	29 (27.1)	
Maternal race	Afro-Caribbean	109 (94.0)	115 (99.1)	0.25
Paternal race ^c^	Afro-Caribbean	109 (94.0)	113 (98.3)	0.82
Maternal education ^d^ (at child’s birth)	Up to high school ^†^	59 (50.9)	87 (77.0)	<0.01
	Beyond high school ^††^	57 (49.1)	26 (23.0)	
Paternal education ^e^ (at child’s birth)	Up to high school ^†^	61 (54.0)	98 (88.3)	<0.01
	Beyond high school ^††^	52 (46.0)	13 (11.7)	
Socioeconomic status (SES)	Car ownership	77 (66.4)	41 (35.3)	<0.01
*GSTP1* ^h^	Ile/Ile	30 (28.3)	26 (24.5)	0.71
	Ile/Val	55 (51.9)	55 (51.9)	
	Val/Val	21 (19.8)	25 (23.6)	
*GSTM1* ^h^	DD ^f^	28 (26.4)	27 (25.5)	0.87
	I/I or I/D ^g^	78 (73.6)	79 (74.5)	
*GSTT1* ^h^	DD ^f^	31 (29.2)	22 (20.8)	0.16
	I/I or I/D ^g^	75 (70.8)	84 (79.2)	
Blood aluminum concentration (µg/L) Arithmetic mean (SD)		30.9 (29.8)	36.9 (40.0)	0.71 **

* *p*-values are based on Wald’s test in conditional logistic regression models that compares the distribution of independent variables between ASD case and TD control groups; ** *p*-value is based on Related-Samples Wilcoxon Signed Rank Test that compares the distribution of blood aluminum concentration between ASD case and TD control groups; ^†^ Up to high school education means attended Primary/Jr. Secondary, and Secondary/High/Technical schools; ^††^ Beyond high school education means attended a Vocational, Tertiary College, or University; ^a^ Maternal age was missing for five TD controls; ^b^ Paternal age was missing for four ASD cases and nine TD controls; ^c^ Paternal race was missing for one ASD case; ^d^ Maternal education was missing for three TD controls; ^e^ Paternal education was missing for three ASD cases and five TD controls; ^f^ DD indicates the null alleles for *GSTT1* and *GSTM1*; ^g^ I/I or I/D indicate the homozygote (I/I) or a heterozygote (I/D) for *GSTT1* and *GSTM1*; ^h^ Results based on 106 matched pairs.

**Table 2:** Associations between dietary consumption and ASD case status using Conditional Logistic Regression (CLR) (232 children or 116 matched pairs).

Exposure Variables	Category		ASD Case *N* (%)	TD Control *N* (%)	Matched OR (MOR)	95% CI for MOR	*p*-Value ^d^
Source of drinking water ^a^	Piped water		110 (94.8)	111 (96.5)	0.67	(0.19, 2.36)	0.53
Source of water for cooking ^b^	Piped water		110 (94.8)	111 (96.5)	0.67	(0.19, 2.36)	0.53
Fruits and vegetables consumption ^c^	Root vegetables	Yam, sweet potato, or dasheen	82 (78.7)	95 (82.6)	0.48	(0.25, 0.93)	0.03
		Carrot or pumpkin	101 (87.1)	113 (98.3)	0.14	(0.03, 0.63)	0.01
	Leafy vegetables	Lettuce	53 (45.7)	73 (63.5)	0.53	(0.33, 0.88)	0.01
		Callaloo, broccoli, or pakchoi	84 (72.4)	108 (93.9)	0.22	(0.10, 0.50)	<0.01
		Cabbage	77 (66.4)	108 (93.9)	0.18	(0.08, 0.40)	<0.01
	Fruits	Tomatoes	72 (62.1)	96 (83.5)	0.29	(0.14, 0.58)	<0.01
		Ackee	68 (58.6)	107 (93.0)	0.05	(0.01, 0.20)	<0.01
		Avocado	31 (26.7)	77 (67.0)	0.18	(0.09, 0.35)	<0.01
		Green banana	82 (70.7)	103 (89.6)	0.28	(0.14, 0.55)	<0.01
		Fried plantains	77 (66.4)	104 (90.4)	0.19	(0.07, 0.48)	<0.01
Seafood consumption	Ate salt water fish		90 (77.6)	104 (89.7)	0.39	(0.18, 0.85)	0.02
	Ate fresh water fish (Pond fish, Tilapia)		50 (43.1)	65 (56.0)	0.56	(0.32, 0.98)	0.04
	Ate sardine, mackerel (Canned fish)		87 (75.0)	107 (92.2)	0.26	(0.11, 0.60)	<0.01
	Ate tuna (Canned fish)		40 (34.5)	50 (43.1)	0.67	(0.38, 1.17)	0.16
	Ate salted fish (Pickled mackerel)		82 (70.7)	106 (91.4)	0.20	(0.08, 0.48)	<0.01
	Ate shellfish (Lobsters, Crabs)		8 (6.9)	16 (13.8)	0.43	(0.17, 1.12)	0.08
	Ate shrimp		24 (20.7)	33 (28.40)	0.64	(0.34, 1.20)	0.16

^a^ Source of drinking water was missing for one TD control; ^b^ Source of water for cooking was missing for one TD control; ^c^ For all variables under fruits and vegetables consumption data were missing for one TD control; ^d^ *p*-values are based on Wald’s test in conditional logistic regression models that compares the distribution of dietary consumption between and ASD case and TD control groups.

**Table 3:** Associations of various independent variables with blood aluminum concentrations based on univariable General Linear Models (116 matched pairs).

Variables	Category		Yes		No		*p*-Value **
			Mean Al * (μg/L)	*N*	Mean Al * (μg/L)	*N*	
Genes ^a^	*GSTT1* (I *) ^b^		22.91	157	23.02	55	0.98
	*GSTM1* (I *) ^b^		25.99	159	22.03	53	0.31
	*GSTP1* (Ile/Ile) ^c^		22.39	56	24.76	156	0.54
	*GSTP1* (Val/Val) ^c^		18.43	46	24.45	166	0.10
	*GSTP1* (Ile/Val) ^c^		24.25	110	21.71	102	0.43
ASD status	Autism Spectrum Disorder		21.49	116	20.95	116	0.78
Child’s age (months)	Age > 48		22.12	191	17.49	41	0.62
Child’s sex	Male		21.19	198	21.39	34	0.96
Socioeconomic status	Own a car		22.35	118	20.11	114	0.44
Maternal age ^d^ (at child’s birth)	≥35 years		22.76	40	20.93	187	0.65
Parental education levels ^e^ (at child’s birth)	At least one of the parents had education beyond high school		23.39	108	18.36	113	0.06
Source of drinking water ^f^	Piped water		20.93	221	26.93	10	0.43
Fruits and vegetables consumption ^g^	Root vegetables	Yam, sweet potato, or dasheen	22.37	177	17.83	54	0.16
		Carrot or pumpkin	21.14	214	22.23	17	0.85
	Leafy vegetables	Lettuce	21.43	126	20.98	105	0.86
		Callaloo, broccoli, or pak choi	21.24	192	21.15	39	0.98
		Cabbage	20.92	185	22.53	46	0.65
	Fruits	Tomatoes	22.06	168	19.14	63	0.38
		Ackee	21.92	175	19.23	56	0.48
		Avocado	24.12	108	18.97	123	0.10
		Green banana	22.10	181	18.30	50	0.22
		Fried plantains	21.71	185	19.36	46	0.55
Seafood consumption	High seafood consumption (more than 6 meals per week)		23.20	82	20.21	150	0.35
	Ate salt water fish		22.04	194	17.46	38	0.20
	Ate fresh water fish (pond fish, tilapia)		20.40	115	22.05	117	0.58
	Ate sardine, mackerel (canned fish)		21.83	194	18.37	38	0.34
	Ate tuna (canned fish)		24.63	90	19.31	142	0.09
	Ate salted fish (pickled mackerel)		21.43	188	20.34	44	0.77
	Ate shellfish (lobsters, crabs)		16.97	24	21.77	208	0.27
	Ate shrimp		20.69	57	21.39	175	0.83

* Mean Al indicates the geometric mean = Exp. [Mean (ln Al)]; ** *p*-values are based on GLMs that compare geometric mean blood aluminum concentrations between children who had the characteristic described (in the “Yes” column) and those who did not (in the “No” column); The “Yes” column includes participants who had the characteristic described for the categories in each variable; The “No” column includes participants who did not have the characteristic described for the categories in each variable; ^a^ Results based on 106 matched pairs; ^b^ I* indicates the homozygote (I/I) or a heterozygote (I/D) for *GSTT1* and *GSTM1*; ^c^ *GSTP1* has three categories (Ile/Ile, Ile/Val, and Val/Val); ^d^ Maternal age was missing for five participants; ^e^ Parental education level was missing for 11 participants; ^f^ Source of drinking water was missing for one participant; ^g^ Fruits and vegetables consumption was missing for one participant.

**Table 4:** Unadjusted and adjusted geometric mean blood aluminum concentrations by *GSTP1* genotypes based on General Linear Models (GLM) that include interaction between *GSTP1* and ASD case status (ASD and TD control) (106 matched pairs).

Models	Gene	(Column A) Genotypes Compared	Referent Genotypes	Group	Unadjusted (μg/L) **			Adjusted (μg/L) ^c^		
					Geometric Mean Al of Children with Genotypes in Column A *	Geometric Mean Al of Children with Referent Genotypes *	*p* ^d^	Geometric Mean Al of Children with Genotypes in Column A *	Geometric Mean Al of Children with Referent Genotypes *	*p* ^d^
Full ^a^	*GSTP1*	Ile/Ile	Ile/Val	TD Control	27.57	26.02	0.81	24.19	23.39	0.89
	*GSTP1*	Ile/Ile	Ile/Val	ASD Case	22.81	22.54	0.96	21.71	23.16	0.77
	*GSTP1*	Ile/Ile	Val/Val	TD Control	27.57	14.00	0.02	24.19	14.60	0.07
	*GSTP1*	Ile/Ile	Val/Val	ASD Case	22.81	25.56	0.69	21.71	24.39	0.68
	*GSTP1*	Ile/Val	Val/Val	TD Control	26.02	14.00	0.01	23.39	14.60	0.05
	*GSTP1*	Ile/Val	Val/Val	ASD Case	22.54	25.56	0.62	23.16	24.39	0.84
Recessive ^b^	*GSTP1*REC	Ile/Ile or Ile/Val	Val/Val	TD Control	26.50	14.01	<0.01	23.75	14.57	0.03
	*GSTP1*REC	Ile/Ile or Ile/Val	Val/Val	ASD Case	22.62	25.62	0.60	22.62	24.38	0.76

* Mean AL indicates the geometric mean = Exp. [Mean (ln AL)]; ** In the univariable GLMs, the independent variables include pairs, ASD status, *GSTP1*, and *GSTP1* interaction with ASD; ^a^ *GSTP1* in the full model has three categories (Ile/Ile, Ile/Val, and Val/Val); ^b^ *GSTP1* (REC) = *GSTP1* in the recessive model has two categories (Val/Val, Ile/Ile or Ile/Val); ^c^ In multivariable GLMs in addition to the variables in the univariable model we adjusted for parental education levels, consumption of root vegetables (yam, sweet potato, or dasheen), avocado, and tuna (canned fish); ^d^ *p*-values are for the comparison of mean blood aluminum concentrations of children with genotypes in “Column A” compared to those with “referent genotypes”, stratified by ASD case status (ASD and TD control), based on CONTRAST option in the SAS program for GLMs as described in the Methods Section.

**Table 5:** Unadjusted and adjusted geometric mean blood aluminum concentrations by ASD status (ASD and TD control) based on General Linear Models (GLM) that includes interaction between *GSTP1* genotypes and ASD case status (ASD and TD control) (106 matched pairs).

Models	(Column A) Group Compared	Referent Group	*GSTP1* Genotypes	Unadjusted Interactive Model (μg/L) **			Adjusted Interactive Model (μg/L) ^c^		
				Geometric Mean Al of Children with Group Compared in Column A *	Geometric Mean Al of Children with Referent Group *	*p* ^d^	Geometric Mean Al of Children with Group Compared in Column A *	Geometric Mean Al of Children with Referent Group *	*p* ^d^
Full ^a^	ASD Case	TD Control	Ile/Ile	22.81	27.57	0.43	21.71	24.19	0.66
	ASD Case	TD Control	Ile/Val	22.54	26.02	0.38	23.16	23.39	0.96
	ASD Case	TD Control	Val/Val	25.56	14.00	0.03	24.39	14.60	0.07
Recessive ^b^	ASD Case	TD Control	Val/Val	25.62	14.01	0.03	24.38	14.57	0.07
	ASD Case	TD Control	Ile/Ile or Ile/Val	22.62	26.50	0.18	22.62	23.75	0.73

* Mean AL indicates the geometric mean = Exp. [Mean (ln AL)]; ** In the univariable GLMs, the independent variables include pairs, ASD status, *GSTP1*, and *GSTP1* interaction with ASD; ^a^ *GSTP1* in the full model has three categories (Ile/Ile, Ile/Val, and Val/Val); ^b^ *GSTP1* (REC) = *GSTP1* in the recessive model has two categories (Val/Val, Ile/Ile or Ile/Val); ^c^ In multivariable GLMs in addition to the variables in the univariable model we adjusted for parental education levels, consumption of root vegetables (yam, sweet potato, or dasheen), avocado, and tuna (canned fish); ^d^ *p*-values are for the comparison of mean blood aluminum concentrations of children with the ASD case status in “Column A” compared to those with the TD control status in “referent group”, stratified by *GSTP1* genotypes , based on CONTRAST option in the SAS program for GLMs as described in the Methods Section.
